# Supplementary material for: Dwarf planet (1) Ceres surface bluing due to high porosity resulting from sublimation
Source: Nat Commun. 2021 Jan 12;12:274. doi: 10.1038/s41467-020-20494-5 (PMC7804090; doi:10.1038/s41467-020-20494-5)
Supplement: Supplementary file 1 — Supplementary Information [file 41467_2020_20494_MOESM1_ESM.pdf]

Material supplementary to:

Dwarf planet (1) Ceres surface bluing due to high porosity resulting from sublimation

Nature Communications

Stefan E. Schröder[1], Olivier Poch[2], Marco Ferrari[3], Simone De Angelis[3], Robin Sultana[2], Sandra M. Potin[2], Pierre Beck[2], Maria Cristina De Sanctis[3], and Bernard Schmitt[2]

[1]Deutsches Zentrum für Luft- und Raumfahrt (DLR), 12489 Berlin, Germany
[2]University Grenoble Alpes, CNRS, Institut de Planétologie et d'Astrophysique de Grenoble (IPAG), 38000 Grenoble, France
[3]Istituto di Astrofisica e Planetologia Spaziali-INAF, 00133 Roma, Italy

Supplementary Note 1. Nontronite sublimation experiment

The sublimation experiment was performed with pure nontronite as a dry run for the experiment with the Ceres analogue material. A sample of nontronite (NaU-1) from the Source Clays Repository of The Clay Minerals Society [1], was ground and sieved as a fine powder (grain size < 36 μm) at the *Istituto di Astrofisica e Planetologia Spaziali-INAF* in Rome, Italy. As opposed to the nontronite in the Ceres analogue material, the pure nontronite did not bear NH_4. It appeared pale yellow to the eye. After spending several hours in an oven at 90°C under medium vacuum to remove the most labile water molecules adsorbed to the phyllosilicates, SHINE acquired a reference spectrum of the nontronite in the CarboNIR chamber under high vacuum and room temperature. When decreasing the pressure from medium to high vacuum, we found that the release of residual adsorbed and interlayer water could suddenly build up a strong gas pressure, leading the nontronite sample to literally explode. The Ceres analogue material never suffered this fate.

On Tuesday 16 April 2019, we prepared the nontronite icy sample as follows. We added 0.700 g nontronite to 70.0 ml ultra-pure water in a glass beaker on a magnetic stirrer to achieve a 1% suspension by weight. The nontronite was easily dispersed and SPIPA-B ice particles were produced without incident using liquid nitrogen. The icy sample appeared pale yellow to the eye. It was transferred to the CarboNIR cryogenic chamber and held at 173 ± 1 K under high vacuum (∼1.5 × 10^{-6} mbar) in a contained coated with black aluminium tape. The ice sublimated over the course of several days, and the sample surface receded towards the bottom of the container while developing small (mm-sized) sublimation pits. A SHINE spectrum was acquired hourly. Initially, the spectra showed strong water ice lines, which gradually disappeared. After 3 days at 173 K, the spectrum of the (now bright yellow) residue did not change appreciably over the hourly intervals. We inferred the absence of water ice below the surface from the duration of the sublimation experiment (by comparison with previous sublimation experiments) and the absence of any motion on the surface of the residue caused by the passing flow of water vapour. However, we were mistaken. We decided to warm the sample to room temperature over the weekend in steps of 2 degrees per hour. On the morning of Monday 22 April, we discovered that the sample had exploded at 215 ± 4 K, likely when the increased gas pressure had exceeded the tensile strength of the residue mantle [2]. The residue had folded over itself outwardly in blanket fashion, and a thin (~1 mm) layer remained in the centre of the container through which the black tape was faintly visible (Supplementary Fig. 1a). After opening the chamber, the residue was retrieved. We found its texture to be fluffy, almost spongy. It would also stick to a spatula and break apart in large pieces, indicating a strong internal cohesiveness. Supplementary Figure 1b shows an optical microscope image of one fragment, which is composed entirely of small spheres of the same size as the original SPIPA-B ice particles.

The sublimation experiment started on the same day at 15:00 h and lasted for 132 hours. Supplementary Figure 2 shows all SHINE spectra acquired during this period. The first spectrum is shown in blue, with the water ice bands at 1.5 and 2.0 μm are clearly visible. The first spectrum of the heating ramp is shown in orange. The last spectrum acquired before the explosion, when the residue was still in a thick (~1 cm) layer, is shown in green. The ice bands reappeared after the explosion, indicating that an ice-rich surface was revealed. The last spectrum, for the thin (~1 mm) layer residue at room

temperature, is shown in red. Water ice lines are absent from both the green and red spectra, indicating that the surface was free of ice. Then the fact that the thick residue is much more reflective than the thin residue over large parts of the spectrum must be due to multiple scattering inside the thick layer. As the thin layer was transparent to some degree, the spectral characteristics of the underlying aluminium tape must be taken into account. Below 2.7 µm, the tape is less reflective than the residue, and therefore acted as a sink for light that passed through the layer. Above 2.7 µm, the residue is darker than the tape, and most of the light that entered the layer did not make it through. The thin layer spectrum may therefore be more representative for the intrinsic reflective properties of the residue material than the thick layer, although the tape's red spectral slope may have affected the residue spectrum towards the visible wavelength range.

With the thickness of the nontronite playing such an important role, assessing the spectral change introduced by the sublimation experiment is not straightforward. We acquired a reference spectrum of the original nontronite powder in a ~0.5 cm deep container. Supplementary Figure 3a compares this "thick" reference spectrum with the last residue spectrum acquired before the explosion (green curve in Supplementary Fig. 2). The ratio spectrum (red curve) indicates that the residue is bluer than the original material below 2.0 µm. After obtaining the results in Supplementary Fig. 2, the need for a thin layer reference spectrum became clear. We prepared a thin (< 1 mm) disk of nontronite by evaporating a drop of nontronite powder suspended in alcohol on (black) aluminium tape while spinning it slowly. The SHADOWS spectro-gonio radiometer acquired a reflectance spectrum of the disk at room temperature and ambient pressure. Supplementary Figure 3b compares this "thin" spectrum with the last residue spectrum acquired after the explosion (red curve in Supplementary Fig. 2). The sublimation residue is markedly bluer than the original material below 2.5 µm. The residue may still have some adsorbed water, because of the presence of absorption bands in the ratio spectrum at 1.9 and 2.9 µm. The slope of the ratio spectrum increases dramatically towards the shortest wavelength, reminiscent of bluing resulting from Rayleigh scattering. Above 2.5 µm, various complex changes in the contributions of H_2O, OH, and possibly CH_x (in the 3.3 - 3.5 µm region), prevent us from drawing any definite conclusions about spectral changes resulting from sublimation. The nature of the bluing suggests that Rayleigh scattering by sub-micron size structures in the porous matrix of the phyllosilicate spheres in the sublimation residue contributes to the reflectance spectrum [2].

Supplementary Note 2. Spectral calibration: Correction for artefacts

The spectra were calibrated with help of measurements of Spectralon® and Infragold® reference surfaces (Labsphere Inc.) that were performed prior to the experiment [3]. In addition, we corrected the spectra of the Ceres analogue material for instrumental artefacts at 0.66 - 0.68 µm and 0.98 - 1.00 µm. The light illuminating the sample is provided by a monochromator, which is equipped with gratings that polarize the light differently with wavelength. In particular, the light produced from 0.40 to 0.66 µm is strongly polarized compared to the light produced at larger wavelengths, because of a change of grating at 0.68 µm. For material with reflectance sensitive to the polarimetric properties of the light (dark samples like the Ceres analogue material), the spectra display a discontinuity between 0.66 µm and 0.68 µm. A second discontinuity is present between 0.98 µm and 1.00 µm. This artefact has a different cause. It is due to the combined effects of the change of detector and the fact that the vertical position

of the sample surface changed compared to the position of the Spectralon reference surface. The vertical position of the original Ceres analogue material was also not exactly that of the Spectralon surface. Reflected light at wavelengths below 0.99 µm is collected by the visible detector whereas light at larger wavelengths is collected by the infrared detector. Both detectors are located on the same arm of the goniometer, but they have different sensitivity across their surface in the "Gognito" configuration. The change of vertical position of the sample induces a shift of position of the light spot as seen by the detectors, creating the artefact.

To correct for these artefacts, we rely on the spectral range from 0.68 to 0.98 µm to represent the true reflectance, because here the polarization of the incoming light is low and the reflected light is collected by the visible detector, which is less sensitive than the infrared detector to changes of position of the light spot. Supplementary Figure 4 illustrates the correction procedure. The artefact between 0.66 and 0.68 µm was corrected by calculating a new value for the reflectance at 0.66 µm by extrapolation, assuming a linear trend in the spectrum from 0.66 µm to 0.72 µm. Then, the reflectance below 0.66 µm was multiplied by the factor equal to the extrapolated reflectance at 0.66 µm divided by its original value. A similar procedure was used to correct for the artefact occurring between 0.98 and 1.00 µm. We obtained a new reflectance at 1.00 µm by extrapolation, assuming a linear trend in the spectrum from 0.96 µm to 0.98 µm, and corrected the reflectance beyond 1.00 µm accordingly.

Supplementary Note 3. Water vapour lines

The spectrum of the sublimation residue of the Ceres analogue material is affected by bands associated with water vapour in the air in the optical path between light source, sample, and detector. In Supplementary Fig. 5 we show the spectra of the sublimation residue (blue) and the original material (black) with a spectrum of water vapour (red). The vapour spectrum was obtained by dividing two reference spectra of the Infragold target, acquired on days with different atmospheric water content. The residue spectrum has absorption features at the location of vapour bands, as indicated by dotted lines. The water vapour bands at 1.4 and 1.9 µm are subtle, but the vapour band complex between 2.5 and 2.9 µm clearly affects the depth of the 2.7 µm absorption band in the sublimation residue spectrum. Therefore, we cannot draw definite conclusions about a change of band depth as a result of the sublimation experiment.

Supplementary Note 4. SEM images of the sublimation residues of nontronite and Ceres analogue material

Here we include scanning electron microscope (SEM) images of the original sample (Supplementary Fig. 6) and sublimation residue (Supplementary Figs. 7-10) of the Ceres analogue material, and the original sample (Supplementary Fig. 11) and sublimation residue (Supplementary Figs. 12-13) of the pure nontronite material.

Supplementary Figures

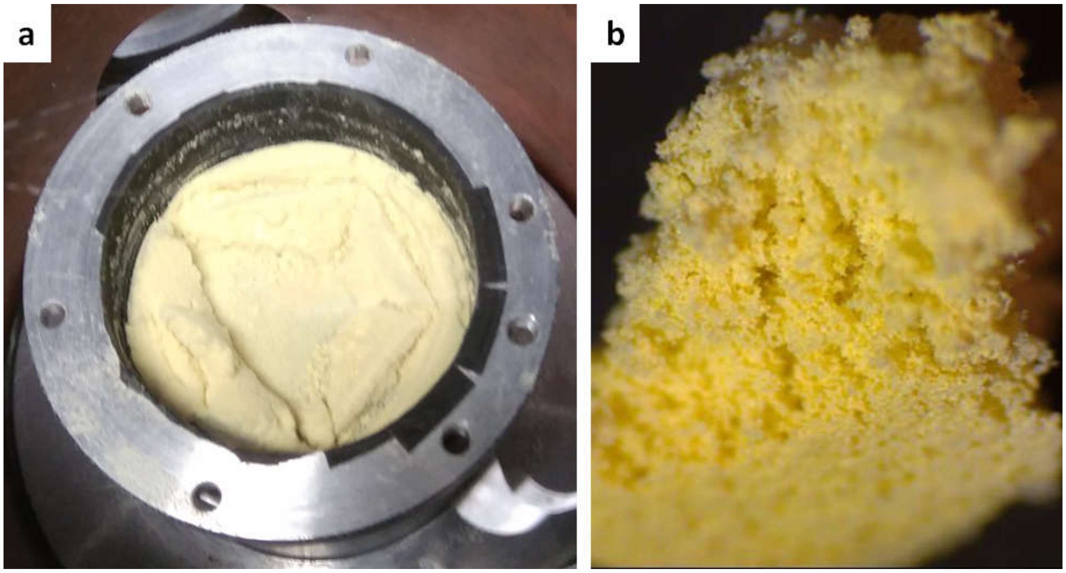

Supplementary Figure 1. The nontronite sublimation residue. (a) The residue inside the cryogenic chamber after the explosion. (b) An optical microscope image of a single fragment of the residue, about 2 mm in size.

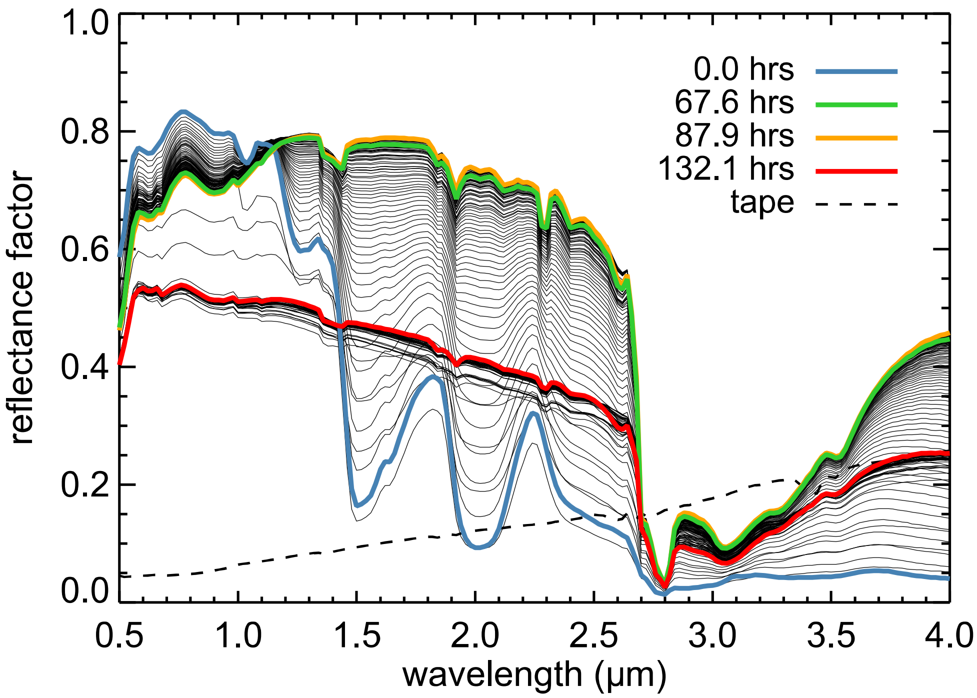

Supplementary Figure 2. Spectral changes during the nontronite sublimation experiment. Spectra of the ice sample under vacuum at 173 K were acquired in approximately hourly intervals. The first and last spectrum of the series are highlighted in blue and red, respectively. The legend displays the time at the start of the acquisition of the spectrum relative to the start of the series. After starting the heating ramp, the sample exploded, leaving a thin layer of residue behind. The green spectrum is the first of the heating ramp, and the orange spectrum is the last complete spectrum acquired before the explosion. The dashed line is the spectrum of the empty container, which is coated with black aluminium tape.

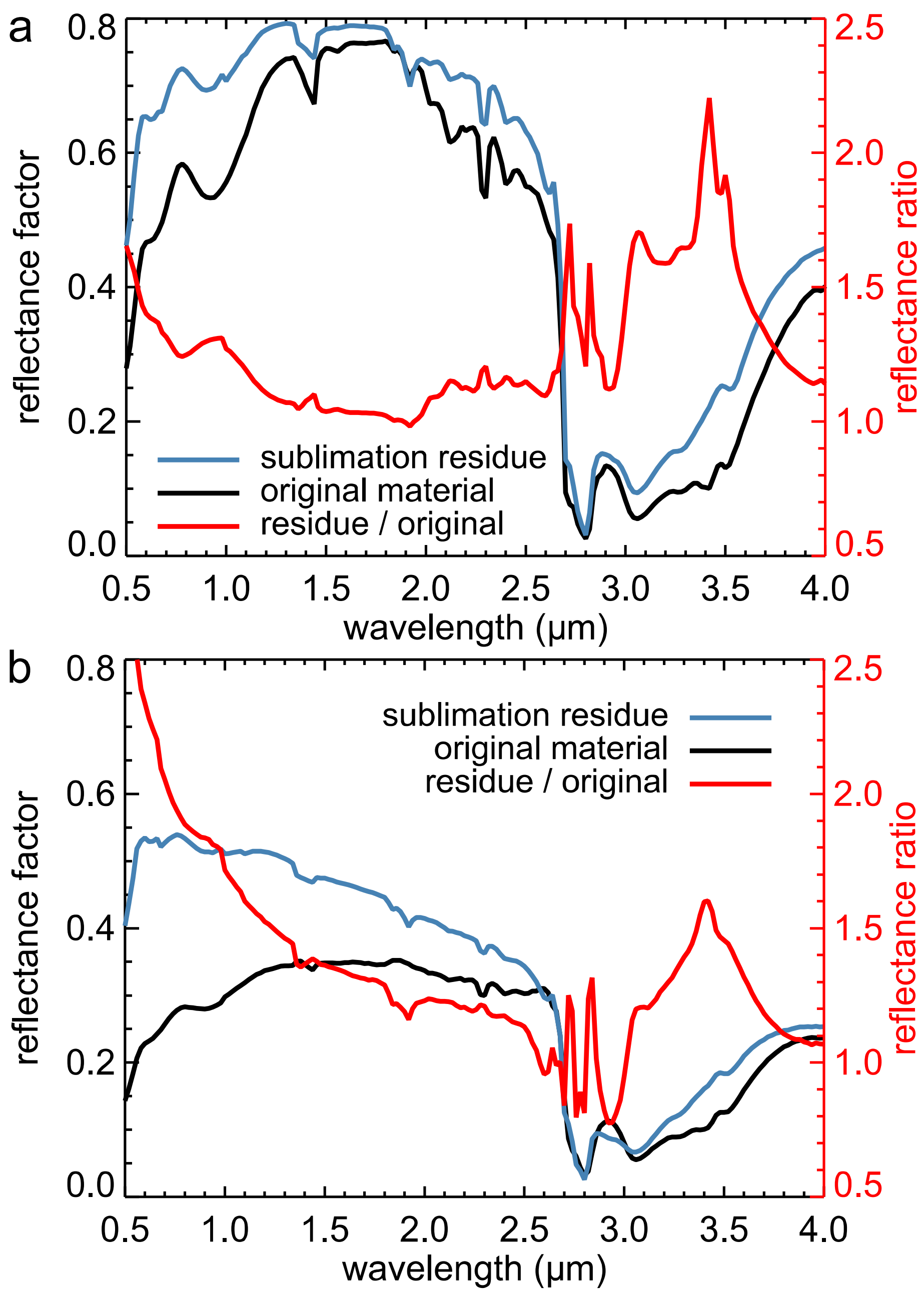

Supplementary Figure 3. Spectral changes induced to the nontronite sample by the sublimation process at the standard geometry of (ι, ε, α) = (0°, 30°, 30°). (**a**) Thick layer. The sublimation residue spectrum was acquired after 87.9 hrs. The original material spectrum was acquired on 16 April. (**b**) Thin layer. The sublimation residue spectrum was acquired after 132.1 hrs. The spectrum of the original material is that of a thin disk on black aluminium tape, acquired on 25 April.

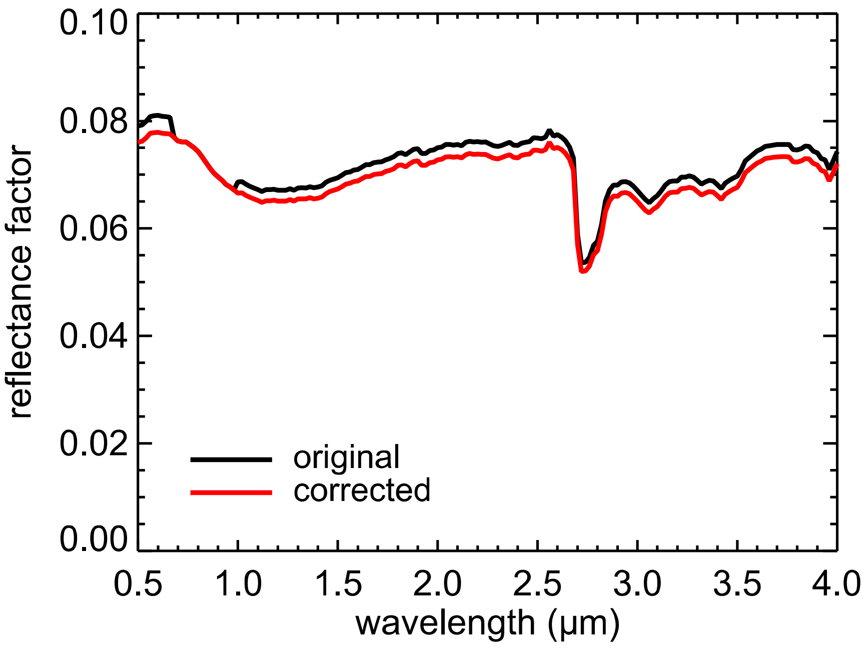

Supplementary Figure 4. Correcting the reflectance spectrum of the Ceres analogue material for instrumental artefacts. The reflectance between 0.68 and 0.98 µm is considered to be trustworthy, and the reflectance values above and below this range are scaled to match.

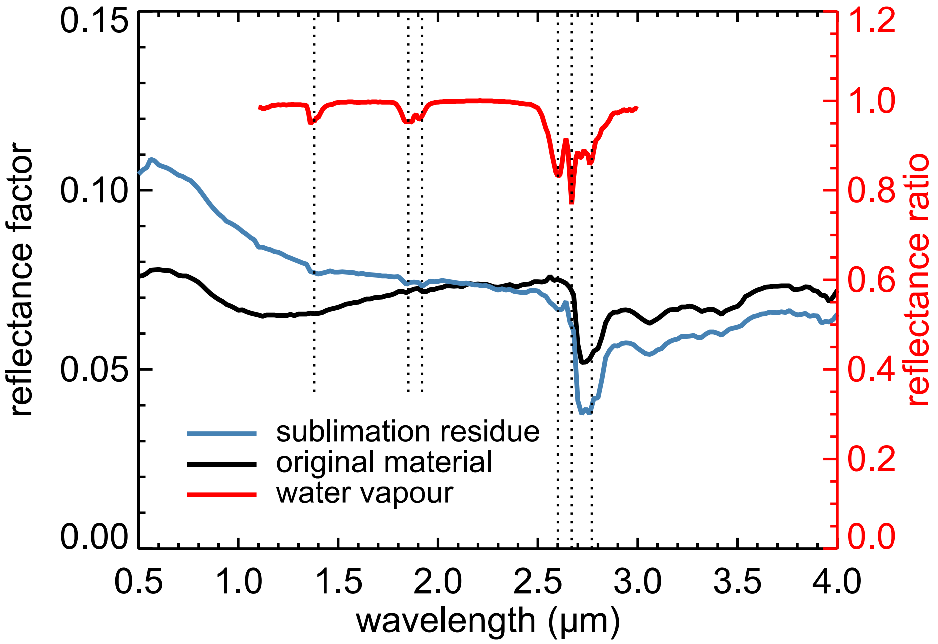

Supplementary Figure 5. Water vapour bands affect the depth of the 2.7 µm band. The sublimation residue spectrum (blue curve) of the Ceres analogue material is affected by water vapour bands. The water vapour spectrum (red curve) was constructed as the ratio of two reference reflectance spectra of the Infragold target that were acquired on days with different atmospheric water content. The dotted lines indicate selected water vapour lines.

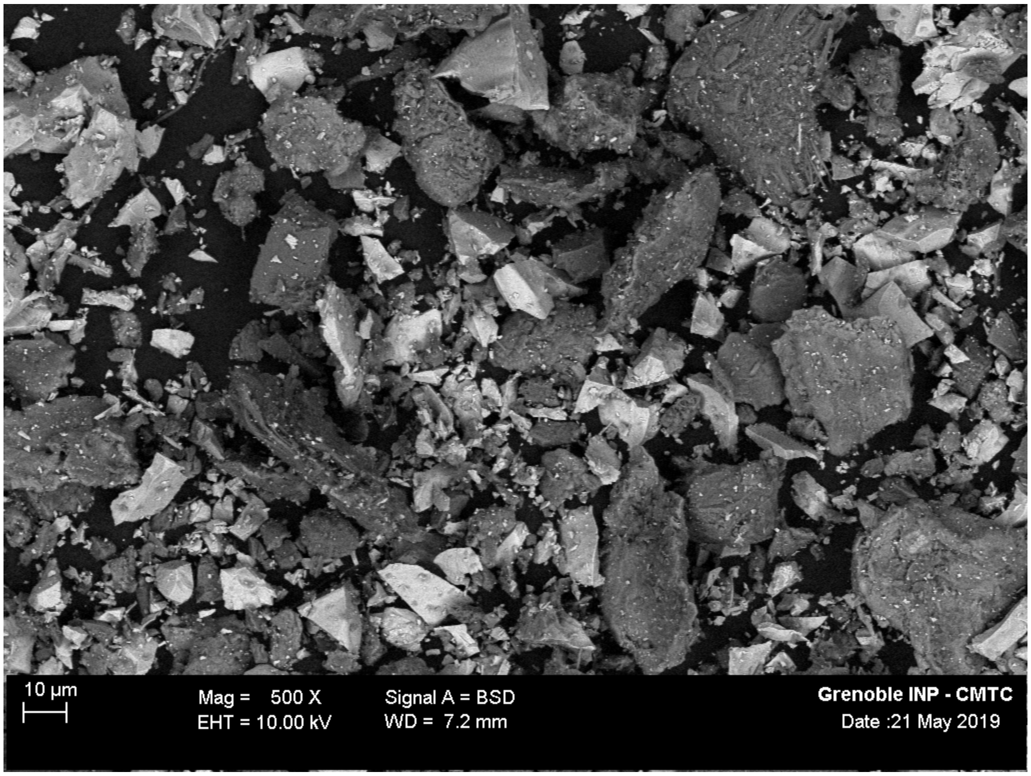

a

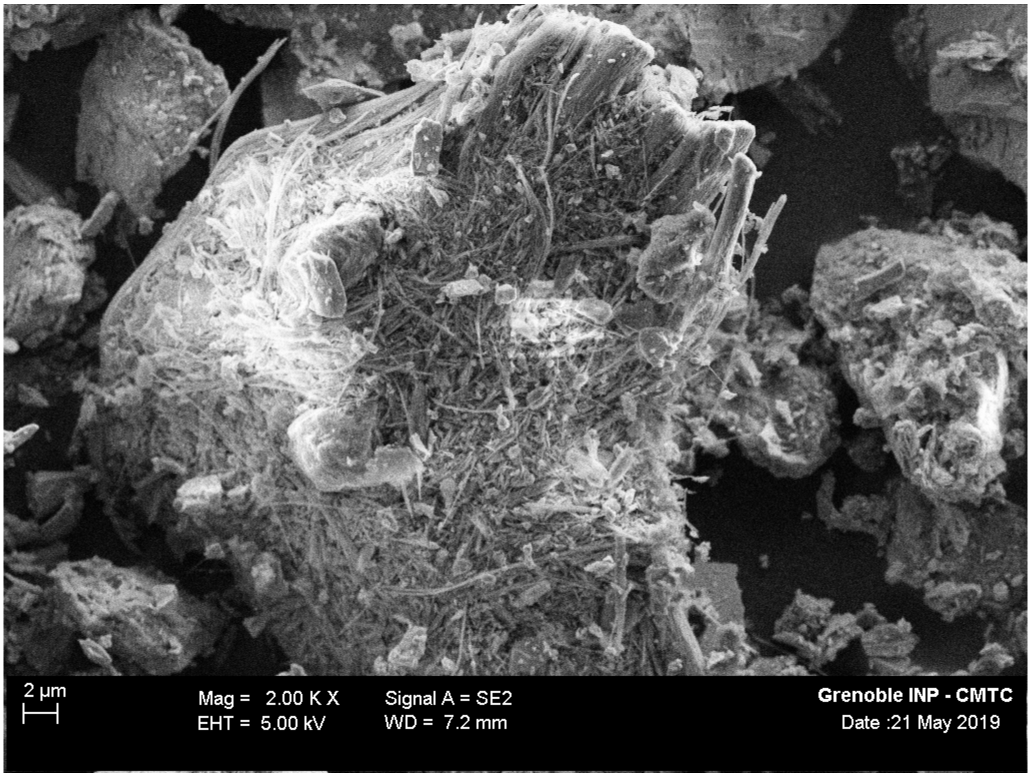

b

Supplementary Figure 6. SEM images of the original Ceres analogue sample. The magnification increases from (a) to (b).

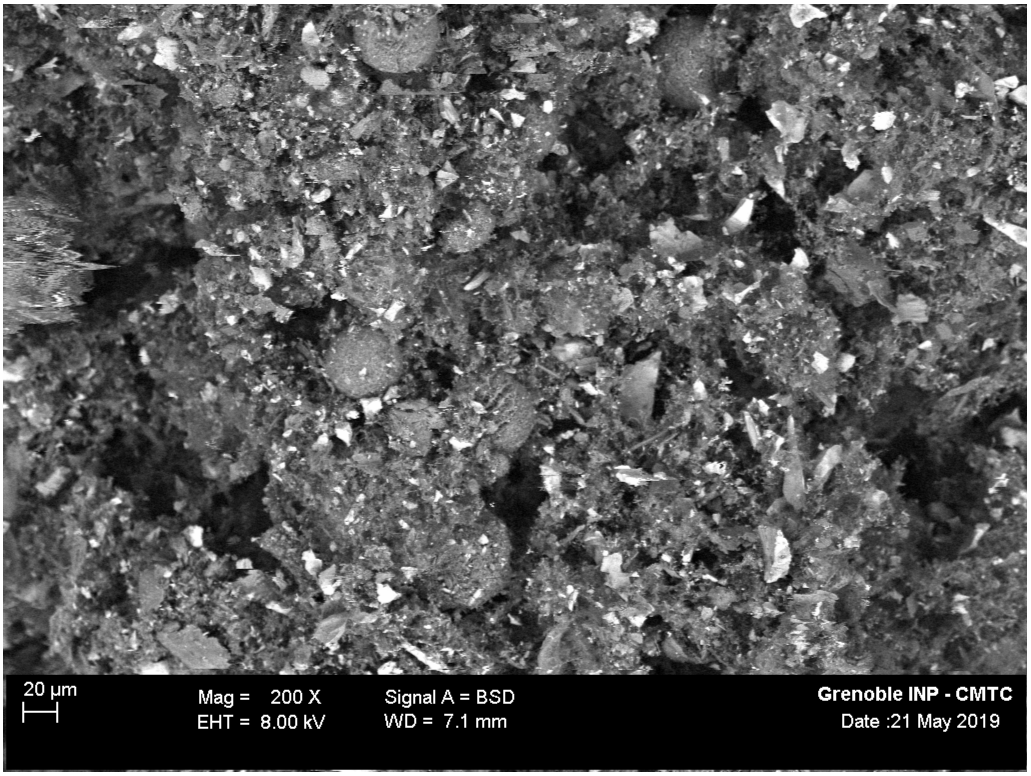

a

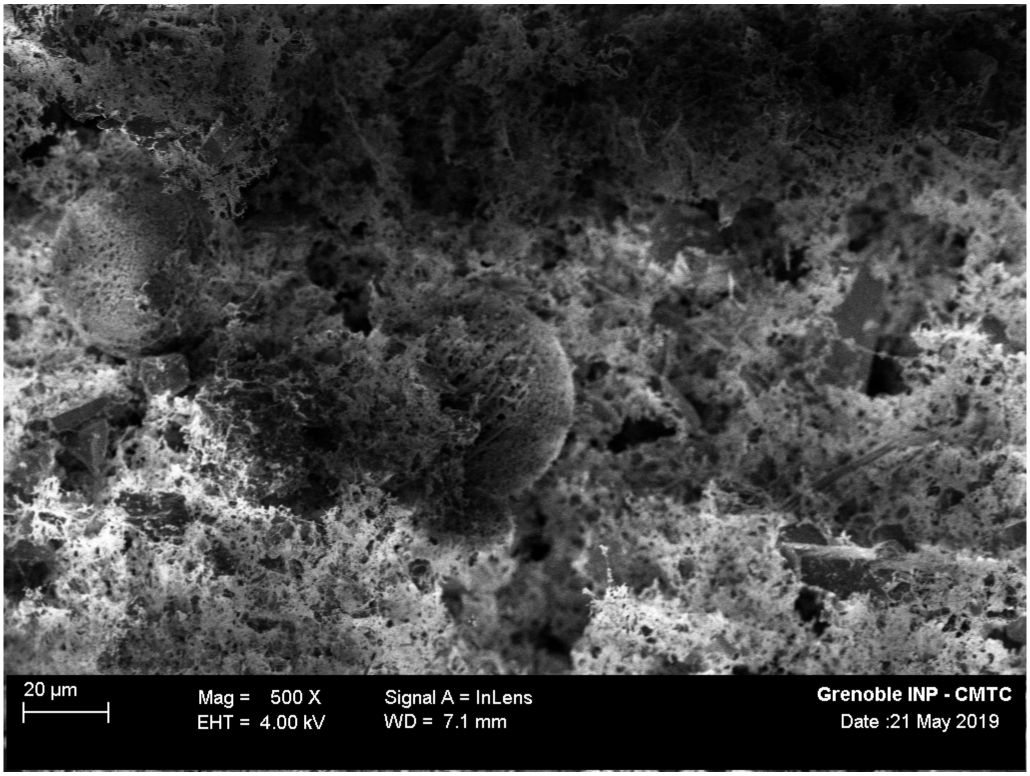

b

Supplementary Figure 7. SEM images of the sublimation residue of the Ceres analogue sample. The magnification increases from (a) to (b).

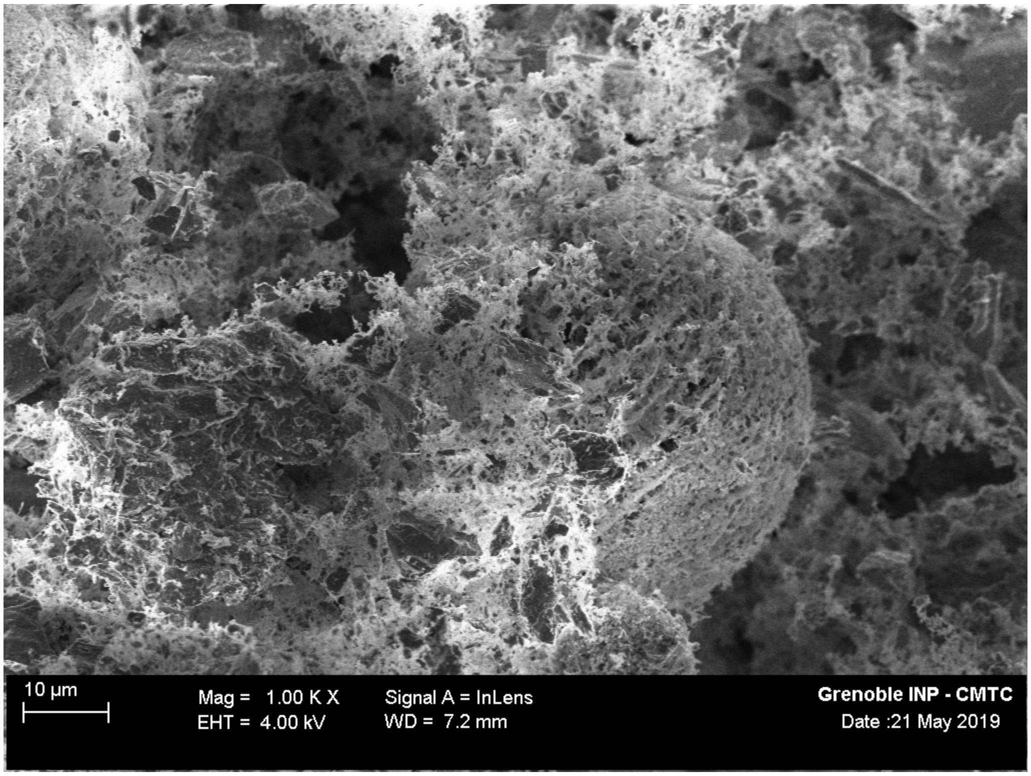

a

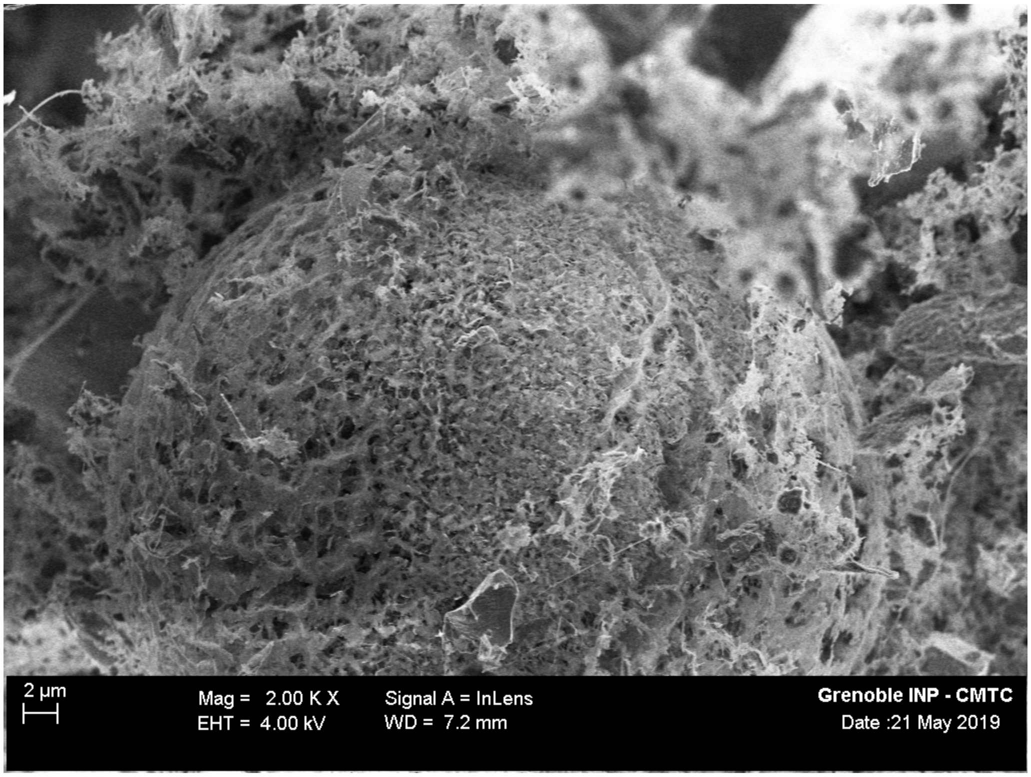

b

Supplementary Figure 8. SEM images of the sublimation residue of the Ceres analogue sample. The magnification increases from (a) to (b).

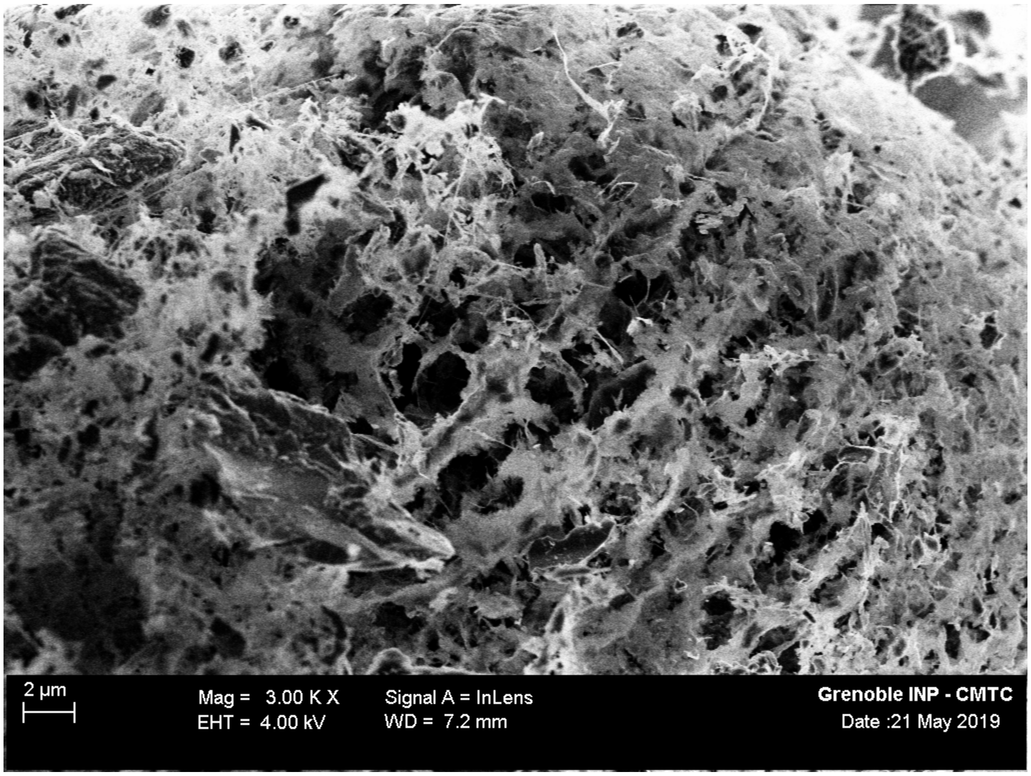

a

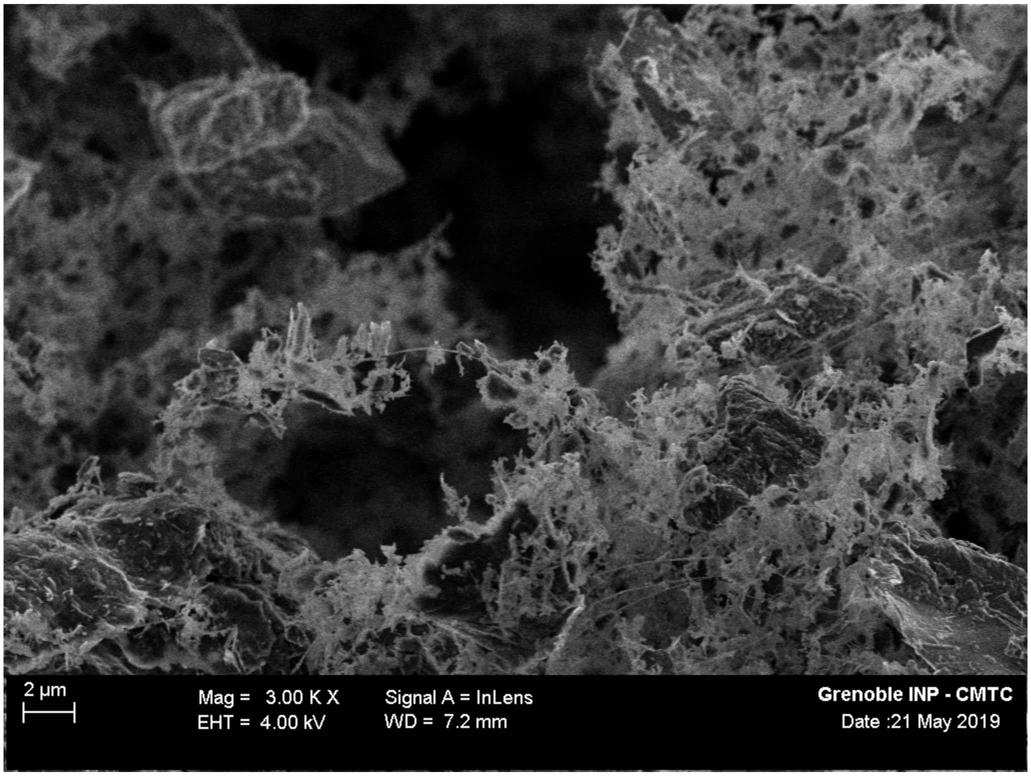

b

Supplementary Figure 9. SEM images of the sublimation residue of the Ceres analogue sample. The magnification increases from (a) to (b). Both images are close-ups of the scene in Supplementary Fig. 8a.

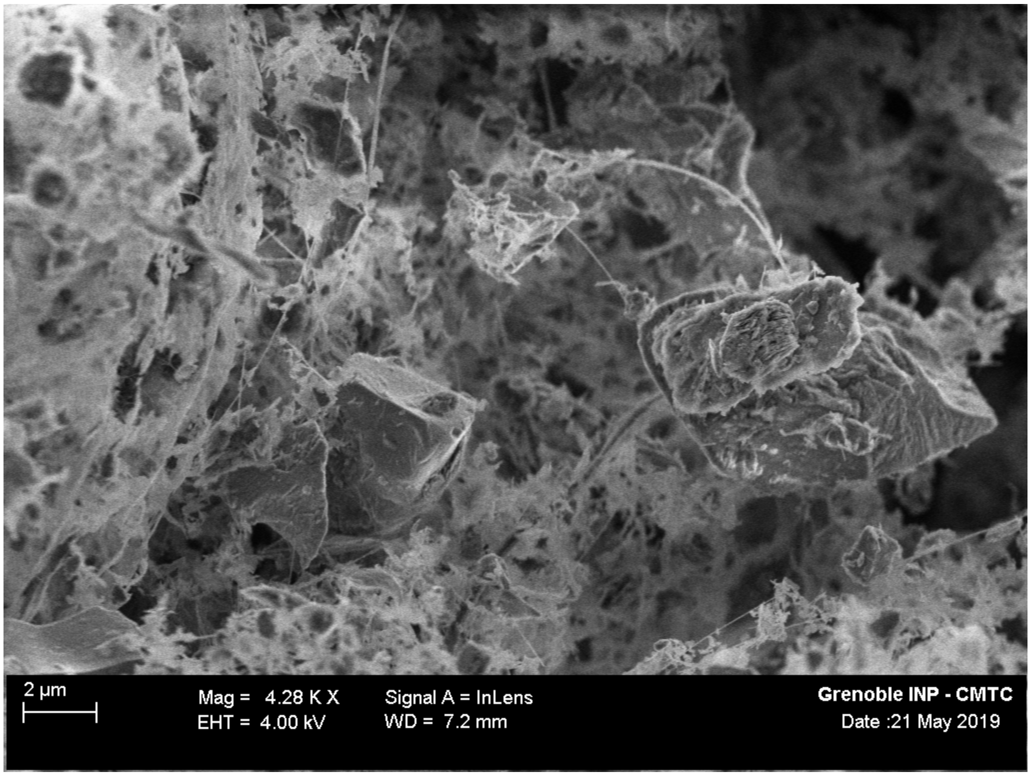

a

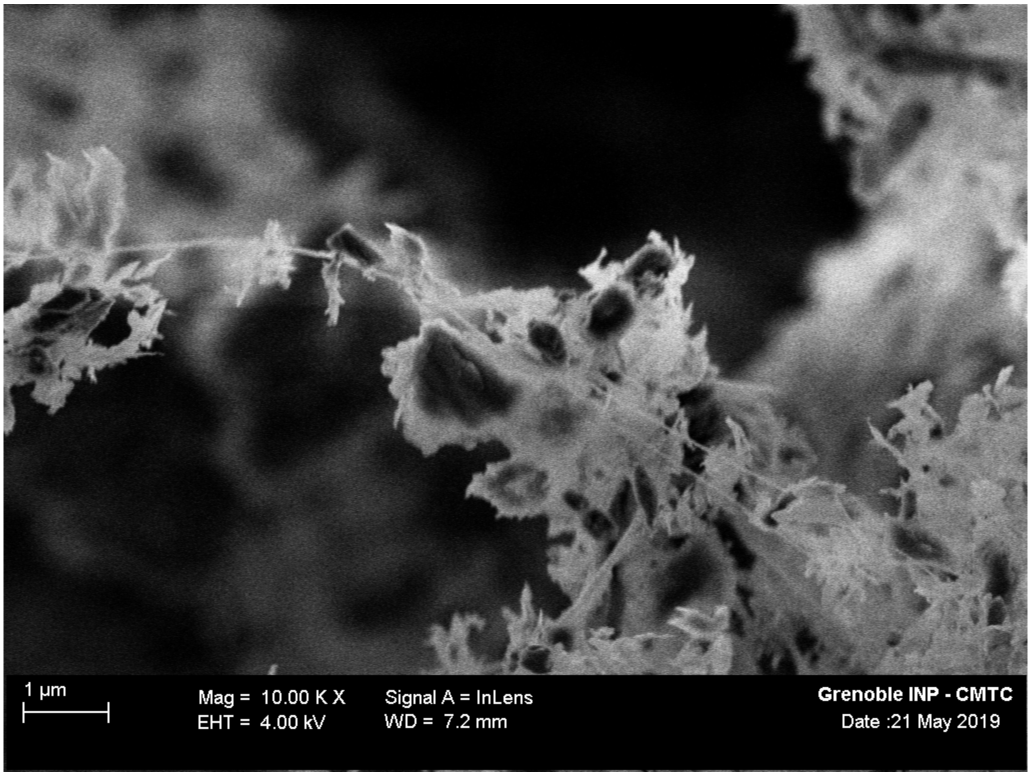

b

Supplementary Figure 10. SEM images of the sublimation residue of the Ceres analogue sample. The magnification increases from (a) to (b). Both images are close-ups of the scene in Supplementary Fig. 9b.

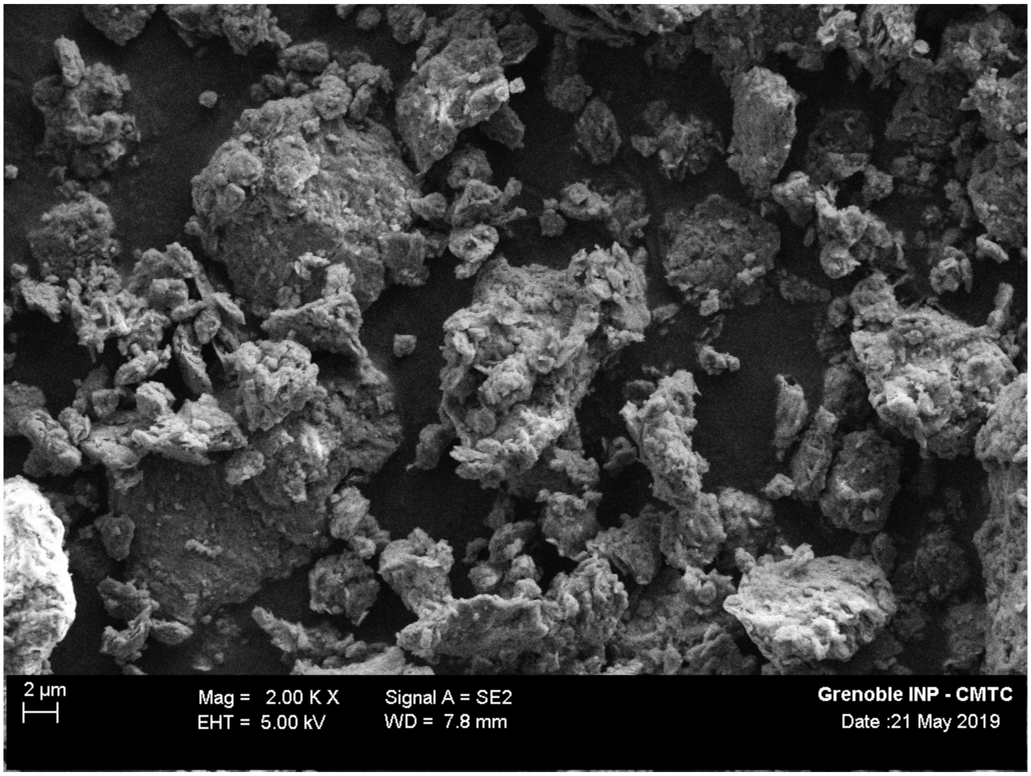

a

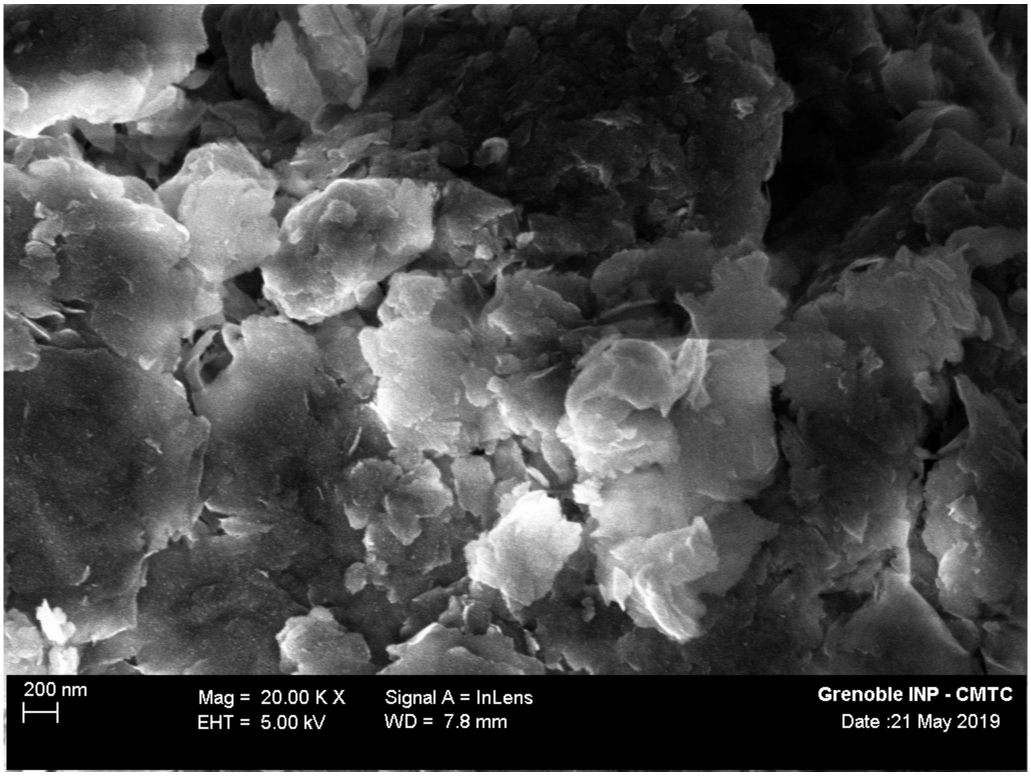

b

Supplementary Figure 11. SEM images of the original pure nontronite sample. The magnification increases from (a) to (b).

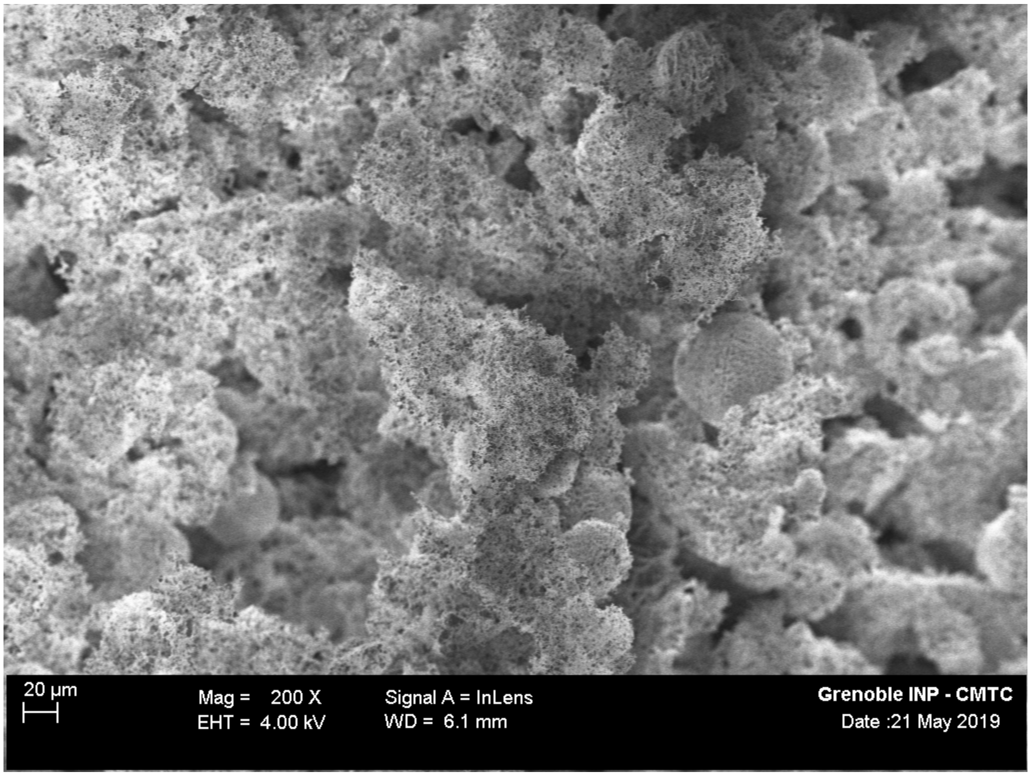

a

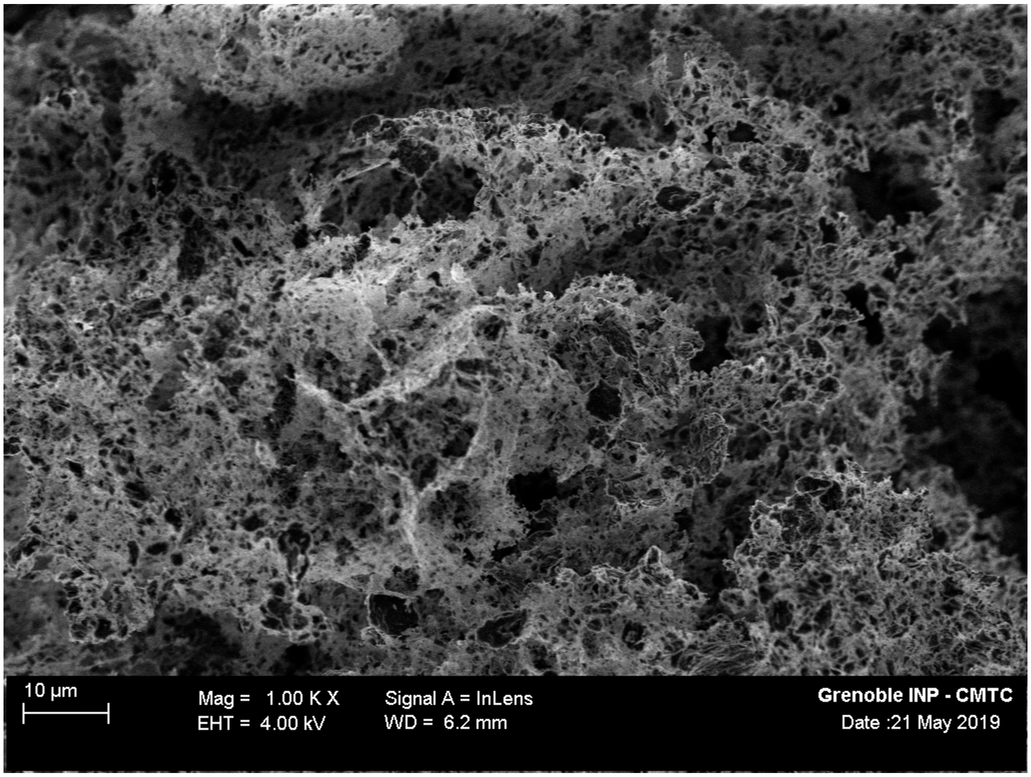

b

Supplementary Figure 12. SEM images of the sublimation residue of the pure nontronite sample. The magnification increases from (a) to (b).

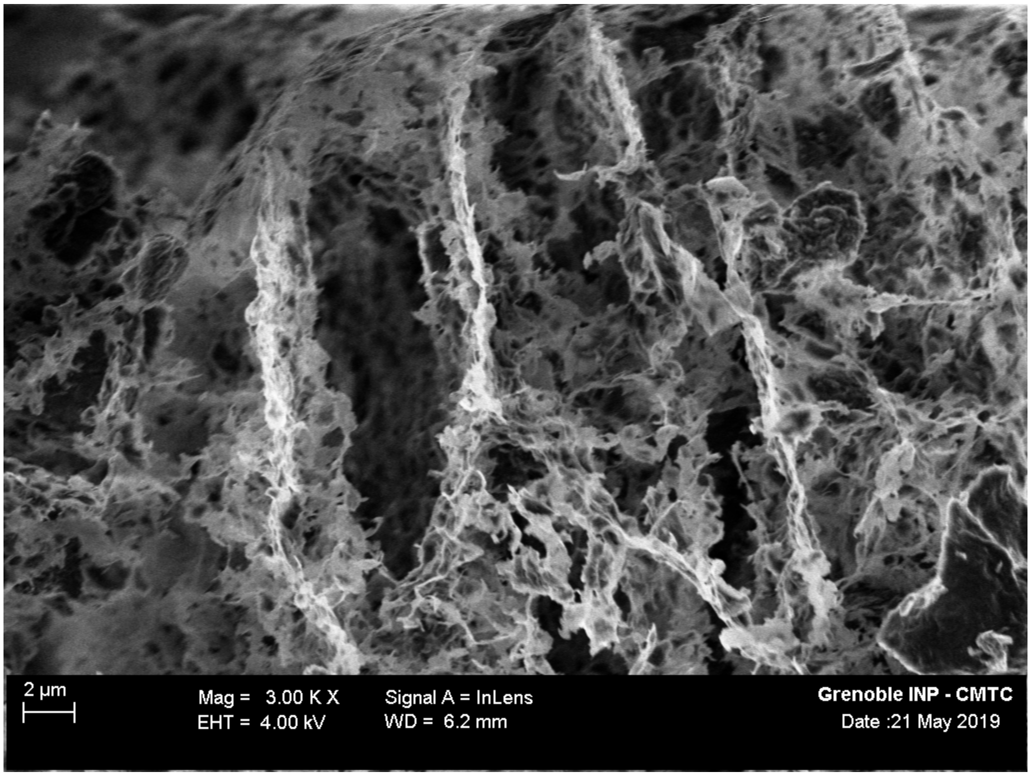

a

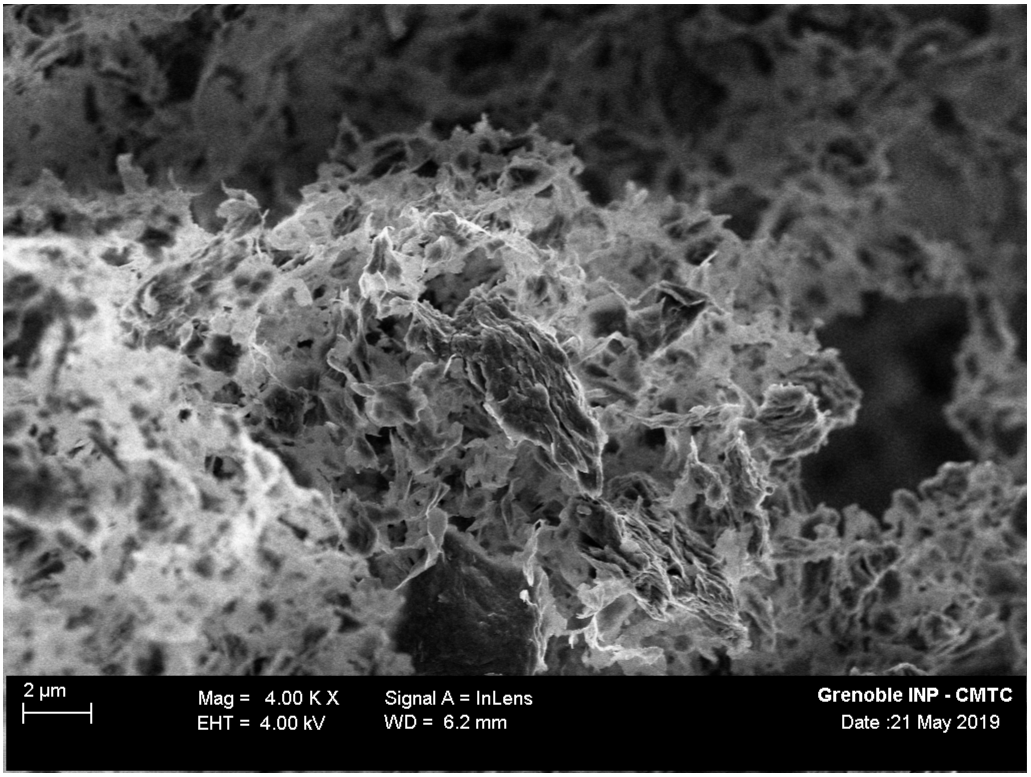

b

Supplementary Figure 13. SEM images of the sublimation residue of the pure nontronite sample. The magnification increases from (a) to (b).

References

[1] J. L. Keeling, M. D. Raven, and W. P. Gates (2000) "Geology and characterization of two hydrothermal nontronites from weathered metamorphic rocks at the Uley graphite mine, South Australia", *Clays and Clay Minerals* **48** (5), 537–548

[2] Olivier Poch, Antoine Pommerol, Bernhard Jost, Nathalie Carrasco, Cyril Szopa, and NicolasThomas (2016) "Sublimation of water ice mixed with silicates and tholins: Evolution of surface texture and reflectance spectra, with implications for comets", *Icarus* **267**, 154-173

[3] Nicolas Bonnefoy (2001) "Développement d'un spectrophoto-goniomètre pour l'étude de la réflectance bidirectionnelle des surfaces géophysiques. Application au soufre et perspectives pour le satellite Io", thèse de l'ED Terre-Univers-Environnement, Université Joseph Fourier, Grenoble
